# Validation of the Portuguese Version of the Modified Harris Hip Score Questionnaire – HHSmBr

**DOI:** 10.1055/s-0044-1792113

**Published:** 2024-12-21

**Authors:** Julia Lavinia Pereira Silva, Debora Pinheiro Lédio Alves, Sebastiana da Costa Figueiredo, Walter Ricioli Junior, Marcelo Cavalheiro de Queiroz, Giancarlo Cavalli Polesello

**Affiliations:** 1 Departamento de Ortopedia e Traumatologia, Faculdade de Ciências Médicas da Santa Casa de Misericórdia de São Paulo (FCMSCSP), São Paulo, SP, Brasil; 2 Grupo de Quadril, Departamento de Ortopedia e Traumatologia, Faculdade de Ciências Médicas da Santa Casa de Misericórdia de São Paulo (FCMSCSP), São Paulo, SP, Brasil

**Keywords:** Harris hip score, hip, surveys and questionnaires, validation study

## Abstract

**Objective** To validate the Portuguese version of the evaluation instrument modified Harris Hip Score.

**Methods** The modified Harris Hip Score went through a validation process for the Portuguese language. We tested the measurement properties of the Brazilian Portuguese version of the modified Harris Hip Score (HHSmBr) on 100 patients (63% females and 37% males) with different hip conditions. Determination of test-retest reliability occurred in 100 participants after an interval of 7 to 14 days. The Cronbach alpha and intraclass correlation coefficient (ICC) evaluated internal consistency and reliability, respectively. The distribution of questions in different categories assessed the floor/ceiling effect. Patients answered the **Western Ontario and McMaster Universities Osteoarthritis Index** ( **WOMAC** ) and the Hip Disability and Osteoarthritis Outcome Score (HOOS) questionnaires to validate estimates.

**Results** The internal consistency of the HHSmBr was 0.724 in the test and 0.706 in the retest. Test-retest reliability was excellent (ICC = 0.80). The floor/ceiling effect only occurred in the pain domain, with scores 23.2% and 12.1% in test and retest, respectively. Comparing the HHSmBr with the WOMAC and HOOS scores, the lowest and highest correlation values were −0.466 and −0.906, respectively, indicating a moderate-to-strong correlation.

**Conclusion** Our study showed that the HHSmBr is a valid and reliable hip-specific assessment questionnaire in Portuguese.

## Introduction

Today, we emphasize outcomes, such as health-related quality of life, functional capacity, pain, and satisfaction scores, because they allow the analysis of the health status and different manifestations of a disease in a person's life. This led to the development and publishing of several instruments, questionnaires, and scores to quantitatively measure these variables since an objective examination is an insufficient indicator of functional, social, and emotional aspects. Patient-reported outcomes are fundamental tools to assess the clinical implication and treatment of musculoskeletal conditions from an individual perspective. 1 2

Several questionnaires evaluate hip conditions, including the Oxford Hip Score (OHS), 3 Lequesne Index of Severity for Osteoarthritis of the Hip (LISOH), 3 Nonarthritic Hip Score, 4 Copenhagen Hip and Groin Outcome Score (HAGOS), 5 and the Harris Hip Score (HHS). The HHS was originally introduced in 1969 to evaluate outcomes from total hip arthroplasty (THA). This questionnaire consists of a score with a maximum of 100 points to assess constructs such as pain, function, deformity, and mobility. Pain and function add up to 44 and 47 points, respectively. Range of motion and deformity yield 5 and 4 points, respectively. Function is subdivided into activities of daily living (14 points) and gait (33 points). A total score lower than 70 points indicates a poor outcome, whereas 70 to 80 points indicate fair, 80 to 90, good, and 90 to 100, excellent outcomes. 6 7

Due to the increase in cases of arthroscopic hip surgeries and the need to evaluate their outcomes, Byrd proposed a modified HHS. This modified version maintains the assessment of pain (44 points) and function (47 points) and multiplies this value by 1.1, a constant, to result in a total score of 100 points. In addition, Byrd eliminated deformity (4 points) and range of motion (5 points) as criteria. 7

Most questionnaires employed in orthopedics are in English. Their use in Brazil requires translation into Portuguese, cross-cultural adaptation, and validation in our population. A previous work translated the HHS and modified HHS questionnaires into Brazilian Portuguese and performed their cross-cultural adaptation, but not their validation. 1 7 8 9 The current study aims to validate the modified HHS assessment instrument for the Brazilian population.

## Materials and Methods

The ethics and research committee approved this cross-sectional study under CAAE 44575121.9.0000.5479. Recommendations from the Consensus-based Standards for the Selection of Health Status Measurement Instruments (COSMIN) checklist and previous studies defined the measurement properties of the Brazilian version of the modified Harris Hip Score (HHSmBr). 10 11

Between May and November 2021, the study included patients over 18 years old screened by the Hip Group from the Department of Orthopedics and Traumatology of our institution with any hip condition regardless of whether or not they had undergone surgical procedure. Subjects with an acute fracture or a history of proximal femur fracture, THA, cognitive deficit, or inability to understand the language were excluded. Patients were informed about the study in person and later contacted by telephone for data collection.

### Procedures

Data collection occurred at 2 different times, with an interval of 7 to 14 days. Two physical therapists applied the questionnaires over the phone and inserted answers and personal data from the subjects on a Google forms (Google LLC, Menlo Park, CA, USA) platform.

In this first stage, patients authorized their participation in the study by signing an informed consent form (according to the Brazilian National Board of Health Resolution No. 510, April 7, 2016). Then, they answered the HHSmBr questionnaire after a brief explanation and the Brazilian version of two other questionnaires (the Western Ontario and McMaster Universities Osteoarthritis Index [WOMAC] and the Hip Disability and Osteoarthritis Outcome Score [HOOS]).

Seven to 14 days later, patients were contacted again by phone to answer the HHSmBr questionnaire (retest) to assess the test-retest reliability. This time between test and retest is short enough to avoid memorization bias, a significant clinical change, or both. 12

The WOMAC is a quality-of-life questionnaire specific for patients with hip and knee osteoarthritis. It has 5 questions about pain (score, 0–20), 2 questions about joint stiffness (0–8), and 17 questions regarding functional limitation (0–68). Each question has a score ranging from 0 to 4. Its minimum score is 0, and the maximum score is 96 points. A higher score indicates a better patient status. 13

The HOOS is a tool to assess patients' opinions about their hip problems and other associated issues. It consists of five subscales: pain, daily living function, sport/recreation function, quality of life, and other hip-related symptoms. The HOOS has 40 questions: 3 are related to hip symptoms and difficulty, 2 assess joint stiffness, 10 refer to hip pain, 17 refer to physical function (ability to move and take care of oneself), 4 address physical function when the patient is more active, and 4 assess the hip-related quality of life. The questions evaluate how the patient felt during the past week. The answers to the questions are standardized, with five alternatives ranging from zero to four points for each question. A score of 100 indicates extreme symptoms, and 0 indicates the absence of symptoms. A normalized score is calculated for each subscale. 14

### Statistical analysis

The sample size was based on previous studies 15 16 17 18 and it is consistent with the literature, which recommends including at least 50 subjects. 19 The Kolmogorov-Smirnov test analyzed data distribution. Other tests verified internal consistency, test-retest reliability, minimum clinically important difference, construct validity, and content validity.

Data are displayed as mean and standard deviation (SD). Statistical analysis was performed using the IBM SPSS Statistics for Windows, version 20.0 (IBM Corp., Armonk, NY, USA), considering a significance level of 5%.

### HHSmBr internal consistency

The Cronbach alpha coefficient assessed the internal consistency of the data. Its maximum value is 1, and internal consistency is adequate if Cronbach alpha coefficient is over 0.7. Higher Cronbach alpha coefficients indicate greater internal consistency. However, Cronbach alpha coefficient must not be higher than 0.95 because it suggests redundant items, the same question asked in a slightly different way, and multicollinearity between items. 18 19

### HHSmBr test-retest reliability

The intraclass correlation coefficient (ICC) test compares the score of the questionnaire applied to the same participants twice. Values for interpretation are the following: < 0.40, low reliability; 0.40 to 0.75, moderate reliability, 0.75 to 0.90, good reliability; > 0.90, excellent reliability. 18 19

### Minimum clinically important difference (MCID)

The MCID was calculated by multiplying the standard error of measurement (SEM) by the square root of 2 and 1.96 (statistical probability with 95% confidence). 18 19

### Validation of the HHSmBr construct

The Pearson correlation coefficient validated the construct by assessing the relationship between HHSmBr and domains from the other questionnaires applied. This coefficient indicates the linearity and strength of the relationship between two data sets but not the agreement between variables. Therefore, it is a complementary analysis to assess the relationship between scores. 18 19

### Distribution of content validity (ceiling/floor effect)

This validity is analyzed from the distribution of questions in different categories. The floor/ceiling effect is present if more than 15% of the participants achieved the lowest or highest possible score with no association with individual effects. 18 19

## Results

The study had 100 participants, with 63% women with a mean age of 50.3 years old (21–86) and 37% men with a mean age of 51.5 years old (23–76). Regarding the educational level, 37% of the patients had completed college, 34% had completed high school, and 29% had incomplete high school.

### HHSmBr internal consistency

The internal consistency of the HHSmBr was good. The Cronbach alpha value was 0.724, indicating good internal consistency as it is above 0.7.

### HHSmBr test-retest reliability

The ICCs for all domains were above 0.80, deemed excellent ( Table 1 ).

**Table 1:** Brazilian Portuguese version of the modified Harris Hip Score test-retest reliability

	ICC	*p* -value
Pain	0.867	< 0.001
Gait	0.967	< 0.001
Daily living	0.840	< 0.001
Function	0.886	< 0.001
Total	0.966	< 0.001

Abbreviations: ICC, intraclass correlation coefficient; HHSmBr, Brazilian Portuguese version of the modified Harris Hip Score.

### Minimum clinically important difference (MCID)

The total MCID value was 6.60 for the test and 7.37 for the retest ( Table 2 ).

**Table 2:** Minimum clinically important difference for HHSmBr

		Mean	Standard deviation	SEM	MCID
Pain	Test	16.26	12.50	1.26	3.48
	Retest	19.7	12.24	1.23	3.41
Gait	Test	18.78	9.62	0.97	2.68
	Retest	18.8	9.68	0.97	2.70
Daily living	Test	8.444	3.49	0.35	0.97
	Retest	8.646	3.53	0.35	0.98
Function	Test	27.22	12.45	1.25	3.47
	Retest	35.74	15.51	1.56	4.32
Total	Test	43.42	23.70	2.38	6.60
	Retest	51.72	26.47	2.66	7.37

Abbreviations: SEM, standard error of measurement; MCID, minimum clinically important difference; HHSmBr, Brazilian Portuguese version of the modified Harris Hip Score.

### Validation of the HHSmBr construct

The HHSmBr construct was validated using the Pearson correlation. The HHSmBr scores correlated with all other scores (WOMAC and HOOS). The lowest and highest correlations were −0.466 and −0.906, respectively. Thus, all correlations were significant, ranging from moderate to strong ( Table 3 ).

**Table 3:** Construct validation for the HHSmBr

			WOMAC	Total HOOS	Stiffness	Pain	Daily living	Sports and recreation	Quality of life
Pain	Test	Corr (r)	−0.845	−0.864	−0.552	−0.855	−0.828	−0.803	−0.809
		*P* -value	< 0.001	< 0.001	< 0.001	< 0.001	< 0.001	< 0.001	< 0.001
	Retest	Corr (r)	−0.770	−0.786	−0.497	−0.789	−0.757	−0.704	−0.736
		*P* -value	< 0.001	< 0.001	< 0.001	< 0.001	< 0.001	< 0.001	< 0.001
Gait	Test	Corr (r)	−0.829	−0.835	−0.605	−0.798	−0.815	−0.699	−0.755
		*P* -value	< 0.001	< 0.001	< 0.001	< 0.001	< 0.001	< 0.001	< 0.001
	Retest	Corr (r)	−0.816	−0.835	−0.580	−0.801	−0.806	−0.720	−0.771
		*P* -value	< 0.001	< 0.001	< 0.001	< 0.001	< 0.001	< 0.001	< 0.001
Daily living	Test	Corr (r)	−0.782	−0.781	−0.566	−0.717	−0.781	−0.661	−0.658
		*P* -value	< 0.001	< 0.001	< 0.001	< 0.001	< 0.001	< 0.001	< 0.001
	Retest	Corr (r)	−0.704	−0.724	−0.466	−0.636	−0.718	−0.694	−0.671
		*P* -value	< 0.001	< 0.001	< 0.001	< 0.001	< 0.001	< 0.001	< 0.001
Function	Test	Corr (r)	−0.855	−0.859	−0.623	−0.814	−0.844	−0.721	−0.764
		*P* -value	< 0.001	< 0.001	< 0.001	< 0.001	< 0.001	< 0.001	< 0.001
	Retest	Corr (r)	−0.821	−0.826	−0.576	−0.765	−0.813	−0.721	−0.748
		*P* -value	< 0.001	< 0.001	< 0.001	< 0.001	< 0.001	< 0.001	< 0.001
HHSmBr	Test	Corr (r)	−0.891	−0.906	−0.617	−0.878	−0.878	−0.801	−0.828
		*P* -value	< 0.001	< 0.001	< 0.001	< 0.001	< 0.001	< 0.001	< 0.001
	Retest	Corr (r)	−0.884	−0.896	−0.602	−0.861	−0.872	−0.796	−0.821
		*P* -value	< 0.001	< 0.001	< 0.001	< 0.001	< 0.001	< 0.001	< 0.001

Abbreviations: WOMAC, Western Ontario and McMaster Universities Osteoarthritis Index; HOOS, Hip Disability and Osteoarthritis Outcome Score; HHSmBr, Brazilian Portuguese version of the modified Harris Hip Score; Corr(r), correlation.

### Distribution of content validity (ceiling/floor effect)

No patient obtained a maximum or minimum score during the HHSmBr test and retest. The pain domain had an index close to 30%, with scores of 23.2 to 12.1% in the test and retest, respectively.

## Discussion

The present study aimed to validate the modified HHS questionnaire previously translated and culturally adapted to the Portuguese language. 7 The HHSmBr version showed acceptable internal consistency to assess patients with different hip conditions, as shown by a Cronbach alpha value of 0.72. This finding corroborates the validation study on the Arabic version of the modified score, with a Cronbach alpha value of 0.7220. It is also consistent with validation studies of the original HHS, which revealed internal consistencies of 0.7, 0.81, and 0.94 for the Turkish, Italian, and Slovenian versions, respectively. 12 16 17

The results showed that the Brazilian version of the questionnaire has proper measurement properties. In addition, its test-retest reliability was excellent, with an ICC of 0.80, ranging from 0.84 to 0.96. The original HHS version presented MCID values ranging from 15.9 to 18 points. 21 Here, the MCID value for HHSmBr goes from 6.60 to 7.37 points. As such, the HHSmBr will help clinical trials and studies evaluating the intervention effect since MCID expresses the clinical perception of improvement by the patient.

The recent literature investigated the HHS's validity by determining its relationship with the outcomes reported by the patient in other questionnaires, such as the Short Form-36 Health Survey (SF-36), Total Functional Score, Nonarthritic Hip Score, and WOMAC. 6 7 12 Our study compared the results of the HHSmBr version with those of the WOMAC and HOOS, previously validated in Portuguese. The HHSmBr presents a high correlation with the WOMAC (r = −0.891) and the HOOS scores (r = −0.906). The same is true for the HHSmBr domains; the domain with the lowest correlation with the WOMAC and HOOS was *daily living* (r = −0.782 and r = −0.781, respectively). 22

Studies for validation of the Arabic version of the modified HHS included samples of 80, 16 103, 17 42, 12 and 183 patients. 20 We determined our sample based on most studies and the COSMIN checklist, which considers that a sample size of 100 patients is excellent, as adopted here. 10 18

This study has some limitations, such as the lack of a specific cognition control in patient inclusion. Although we considered the educational level alone, no comprehension difficulties in answering the questions were noticed and/or reported by patients. This finding indicates that the cognitive variable may not have significantly interfered with the results.

Even though the modified HHS is a self-report questionnaire, the logistics of the service made this application model unfeasible. Thus, we decided to apply the questionnaire over the phone as in previous studies. 23 24 25 In addition, we ensured that the same examiner applied the test and retest, following the same method by phone, as shown by the positive results obtained in this study. Our data demonstrate that this is an effective and viable method of applying questionnaires.

## Conclusion

Our study showed that the HHSmBr is a valid and reliable questionnaire in Portuguese.
