# Supplementary material for: Validation of the Portuguese Version of the Modified Harris Hip Score Questionnaire – HHSmBr
Source: Rev Bras Ortop (Sao Paulo). 2024 Dec 21;59(6):e944–9. doi: 10.1055/s-0044-1792113 (PMC11663059; doi:10.1055/s-0044-1792113)
Supplement: Related texts

# Research article: Validação do questionário Harris Hip Score modificado para a língua portuguesa – HHSmBr

**Keywords:** estudo de validação, inquéritos e questionários, quadril, qualidade de vida

## Abstract

**Objetivo** Validar o instrumento de avaliação *Harris Hip Score* modificado para a língua portuguesa (HHSmBr).

**Métodos** Foi realizado o processo de validação para a língua portuguesa. As propriedades de medição do HHSmBr foram testadas em 100 pacientes (63% mulheres e 37% homens) com diferentes patologias do quadril. A confiabilidade teste-reteste foi aplicada nos 100 participantes com intervalo de 7 a 14 dias. Para avaliar a consistência interna utilizou-se o alfa de Cronbach e para a confiabilidade o coeficiente de correlação intraclasse (CCI). O efeito piso/teto foi avaliado a partir da distribuição de perguntas em diferentes categorias. Os pacientes responderam o **Western Ontario and McMaster Universities Osteoarthritis Index** (WOMAC) e o *Hip Disability and Osteoarthritis Outcome* *Score* (HOOS) para validação da estimativa.

**Resultados** A versão brasileira do HHSm mostrou consistência interna de 0,724 no teste e de 0,706 no reteste e confiabilidade teste-reteste (CCI = 0,80). O efeito piso/teto só ocorreu no domínio dor, com escores de 23,2% e 12,1% no teste e reteste, respectivamente. Ao correlacionar os escores do HHSmBr com os demais escores dos questionários citados, verificou-se que a menor correlação foi de −0,466 e a maior de −0,906 indicando correlação moderada a forte.

**Conclusão** O presente estudo mostrou que o HHSmBr é um questionário de avaliação específica do quadril válido e confiável para ser usado na língua portuguesa.

## Introdução

Atualmente, desfechos como qualidade de vida relacionada à saúde, capacidade funcional, e escalas de dor e satisfação têm sido enfatizados por possibilitarem análise da situação de saúde e diferentes manifestações da doença na vida do indivíduo. Como consequência, foram desenvolvidas e publicadas uma variedade de instrumentos, questionários e escalas que visam mensurar quantitativamente essas variáveis, já que o exame objetivo é um indicador escasso para avaliação de aspectos funcionais, sociais e emocionais. Os resultados relatados pelo paciente são ferramentas importantes para avaliar a implicação clínica das condições musculoesqueléticas e do tratamento sob a perspectiva do paciente. 1 2

Vários questionários têm sido desenvolvidos para avaliar as afecções do quadril, tais como *Oxford Hip Score* (OHS), 3 *Lequesne Index of Severity for Osteoarthritis of the Hip* (LISOH), 3 *Nonarthritic Hip Score* , 4 *Copenhagen Hip and Groin Outcome Score* (HAGOS) 5 e o *Harris Hip Score* (HHS) *,* que foi desenvolvido originalmente em 1969 para avaliar os resultados da artroplastia total de quadril (ATQ). Esse questionário consiste em uma escala com máximo de 100 pontos, incluindo a avaliação de construtos como dor, função, deformidade e mobilidade. Dor e função apresentam 44 e 47 pontos possíveis, respectivamente; amplitude de movimento e deformidade, 5 e 4 pontos, respectivamente. A função foi subdividida em atividades de vida diária (14 pontos) e marcha (33 pontos). Uma pontuação total menor que 70 pontos equivale a um resultado ruim, 70 a 80 razoável, 80 a 90 bom e de 90 a 100 excelente. 6 7

Devido ao aumento dos casos de cirurgias artroscópicas no quadril e a necessidade de avaliação dos seus resultados, Byrd propôs a modificação do HHS (HHS modificado [HHSm]), mantendo a avaliação da dor (44 pontos) e função (47 pontos) e, por fim, a multiplicação pela constante 1,1, resultando em um escore total possível de 100 pontos. Os critérios de deformidade (4 pontos) e amplitude de movimento (5 pontos) foram eliminados. 7

A maioria dos questionários usados em ortopedia foram desenvolvidos em língua inglesa, e para serem usados no Brasil necessitam ser traduzidos para a língua portuguesa, adaptados transculturalmente e validados para a nossa população. Em trabalho anterior, os questionários HHS e HHSm foram traduzidos e adaptados transculturalmente para o português do Brasil, porém não foram validados. 1 7 8 9 O objetivo deste estudo é validar o instrumento de avaliação HHSm para a população brasileira.

## Materiais e Métodos

Este estudo transversal foi aprovado pelo comitê de ética e pesquisa da instituição na qual foi realizado, sob o CAAE: 44575121.9.0000.5479, e as propriedades de medida do HHSm foram definidas de acordo com as recomendações da lista de verificação do *Consensus-based Standards for the Selection of Health Status Measurement Instruments* (COSMIN) e estudos prévios. 10 11

Entre maio e novembro de 2021, foram selecionados para o estudo pacientes acima de 18 anos, triados pelo grupo de quadril do Departamento de Ortopedia e Traumatologia de nossa instituição que apresentassem alguma afecção no quadril independente de terem sido submetidos a procedimento cirúrgico ou não. Foram excluídos indivíduos com fratura aguda ou histórico de fratura da região proximal do fêmur, ATQ, déficit cognitivo ou incapacidade de entender o idioma. Os pacientes foram esclarecidos sobre o estudo presencialmente e contatados posteriormente por via telefônica para a coleta dos dados.

## Procedimentos

A coleta de dados foi realizada em dois tempos diferentes, com um período de intervalo de 7 a 15 dias. A aplicação dos questionários foi realizada por dois fisioterapeutas via ligação telefônica, cujas respostas dos questionários e dados pessoais dos indivíduos foram homologadas na plataforma *Google forms* (Google LLC, Menlo Park, CA, EUA).

Nesta primeira etapa os pacientes autorizaram a participação no estudo por meio do termo de consentimento livre e esclarecido (de acordo com a Resolução n° 510, de 7 de abril de 2016). Em seguida, o HHSm foi administrado após uma breve explicação sobre o mesmo e então foram também administrados outros dois questionários na sua versão brasileira, o Western Ontario and McMaster Universities (WOMAC) e o *Hip Disability and OsteoarthritisOutcome Score* (HOOS).

Após o período de 7 a 14 dias foi realizado um novo contato com o paciente por via telefônica e aplicado o questionário HHSm (reteste) para avaliar a confiabilidade do teste e reteste. Esse tempo entre teste e reteste é considerado suficientemente curto para evitar viés de memorização e/ou qualquer alteração significativa no quadro clínico apresentado. 12

O WOMAC é um questionário de qualidade de vida, específico para a avaliação de pacientes com osteoartrite de quadril e joelho, consiste em 5 perguntas sobre dor (escore de 0–20), 2 perguntas sobre rigidez articular (0–8) e 17 sobre limitação funcional (0–68), cada pergunta com pontuação variando de 0 a 4. Seu escore mínimo é de 0 pontos e máximo de 96 pontos e quanto maior a pontuação melhor o quadro do paciente. 13

O HOOS foi desenvolvido como ferramenta de avaliação da opinião dos pacientes sobre seus problemas de quadril e outros problemas associados e é composto por cinco subescalas: dor, função de vida diária, função em esporte/recreação, qualidade de vida e outros sintomas relacionados ao quadril. São 40 questões; 3 relacionadas a sintomas e dificuldade do quadril; 2 dizem respeito à quantidade de rigidez articular sentida pelo paciente; 10 se referem à dor no quadril; 17 dizem respeito à função física (capacidade de se movimentar e cuidar de si mesmo); 4 são relacionadas à função física quando o paciente está mais ativo; e 4 se referem à qualidade de vida relacionada ao quadril. As questões avaliam como o paciente se sentiu durante a última semana. As respostas das perguntas são padronizadas, são 5 alternativas que variam pontuação de 0 a 4 pontos cada questão, nas quais, uma pontuação de 100 indica sintomas extremos e 0 indica a ausência de sintomas. A pontuação normalizada é calculada para cada subescala. 14

## Análise estatística

A amostra do estudo foi baseada em estudos anteriores, 15 16 17 18 e está de acordo com a literatura, que recomenda incluir pelo menos 50 indivíduos. 19 O teste de Kolmogorov-Smirnov foi aplicado para analisar a distribuição dos dados e foram realizados também testes para verificar a consistência interna, confiabilidade teste-reteste, valor da diferença mínima clinicamente importante e validade de construto, envolvendo também a validade de conteúdo.

Os dados estão expostos como média e desvio padrão (SD). A análise estatística foi realizada pelo programa IBM SPSS Statistics for Windows, versão 20 (IBM Corp., Armonk, NY, EUA), considerando nível de significância de 5%.

## Consistência interna do HHmBr

Para avaliar a consistência interna dos dados foi utilizado o coeficiente alfa de Cronbach. Essa estatística tem o máximo valor de 1 e a consistência é considerada adequada se o valor estiver acima de 0,7, sendo que quanto maior o seu valor, maior é a consistência interna dos dados. Porém, seu valor não deve ser superior a 0,95, pois indica itens de redundância ou a mesma pergunta feita de maneira ligeiramente diferente e multicolinearidade entre os itens. 18 19

## Confiabilidade entre teste e reteste do HHmBr

Foi utilizado o teste de coeficiente de correlação intraclasse (CCI), que compara o escore do questionário aplicado aos mesmos participantes duas vezes. Os valores para a interpretação são: < 0,40 baixa confiabilidade, 0,40 a 0,75 moderada confiabilidade e 0,75 a 0,90 boa confiabilidade e maior que > 0,90 excelente confiabilidade. 18 19

## Valor da diferença Mínima Clinicamente Importante (DMCI)

Para calcular o DMCI, foi utilizado o cálculo do EPM (erro padrão de medida), que consiste na multiplicação do EPM pela raiz quadrada de 2 e também por 1,96 (probabilidade estatística com 95% de confiança). 18 19

## Validação de construto do HHmBr

O coeficiente de correlação de Pearson foi utilizado para realizar a validação de construto por meio da relação do HHSmBr com os domínios dos demais questionários aplicados. Este indica a linearidade e a força da relação entre dois conjuntos de dados, mas não é capaz de indicar a concordância entre as variáveis. Portanto, é uma análise complementar para avaliar a relação dos escores. 18 19

## Distribuição da Validade de Conteúdo (Piso/Teto)

É analisada a partir da distribuição de perguntas em diferentes categorias. O efeito piso/teto é considerado se mais de 15% dos participantes atingiram o menor ou o maior escore possível e não estão relacionados a efeitos individuais. 18 19

## Resultados

Participaram do estudo um total de 100 pacientes 63% mulheres com média de idade de 50,3 anos (21–86) e 37% homens com média de idade de 51,5 anos (23–76). Em relação ao nível de escolaridade, 37% dos pacientes possuíam o ensino superior completo, 34% o ensino médio completo e 29% o ensino médio incompleto.

## Consistência interna do HHmBr

A consistência interna do HHSmBr obteve bons resultados. O valor do alfa de Cronbach foi de 0,724, o que indica uma boa consistência interna, já que consideramos bons os valores acima de 0,7.

## Confiabilidade entre Teste e Reteste do HHmBr

Verificou-se que os CCIs obtidos em todos os domínios foram acima de 0,80, sendo considerado excelente ( Tabela 1 ).

**Tabela 1:** Confiabilidade entre teste de reteste do HHSmBr

	ICC	Valor de *p*
Dor	0,867	< 0,001
Marcha	0,967	< 0,001
Dia a dia	0,840	< 0,001
Função	0,886	< 0,001
Total	0,966	< 0,001

Abreviações: ICC, índice de coeficiente de correlação; HHSmBr, versão em Português do Brasil do Harris Hip Score modificado Hip Score modificado.

## Diferença Mínima Clinicamente Importante (DMCI)

O valor total do DMCI foi de 6,60 no teste e em 7,37 no reteste ( Tabela 2 ).

**Tabela 2:** Diferença mínima clinicamente importante do *HHSmBr*

		Média	Desvio padrão	EPM	DMCI
Dor	Teste	16,26	12,50	1,26	3,48
	Reteste	19,7	12,24	1,23	3,41
Marcha	Teste	18,78	9,62	0,97	2,68
	Reteste	18,8	9,68	0,97	2,70
Dia a dia	Teste	8,444	3,49	0,35	0,97
	Reteste	8,646	3,53	0,35	0,98
Função	Teste	27,22	12,45	1,25	3,47
	Reteste	35,74	15,51	1,56	4,32
Total	Teste	43,42	23,70	2,38	6,60
	Reteste	51,72	26,47	2,66	7,37

Abreviações: EPM, erro padrão de medida; DMCI, diferença mínima clinicamente importante; HHSmBr, versão em português do Brasil do Harris Hip Score modificado.

## Validação de construto do HHmBr

Para validação de construto do HHSmBr por meio da correlação de Pearson, verificou-se que os escores do HHSmBr apresentaram correlação com todos os demais escores (WOMAC e HOOS), sendo a menor correlação de −0,466 e a maior de −0,906. Assim, todas as correlações foram significantes, sendo moderadas e fortes ( Tabela 3 ).

**Tabela 3:** Validação de construto do HHSmBr

			Womac	HOOS total	Rigidez	Dor	Dia a dia	Esporte e lazer	Qualidade de vida
Dor	Teste	Corr (r)	−0,845	−0,864	−0,552	−0,855	−0,828	−0,803	−0,809
		Valor de *p*	< 0,001	< 0,001	< 0,001	< 0,001	< 0,001	< 0,001	< 0,001
	Reteste	Corr (r)	−0,770	−0,786	−0,497	−0,789	−0,757	−0,704	−0,736
		Valor de *p*	< 0,001	< 0,001	< 0,001	< 0,001	< 0,001	< 0,001	< 0,001
Marcha	Teste	Corr (r)	−0,829	−0,835	−0,605	−0,798	−0,815	−0,699	−0,755
		Valor de *p*	< 0,001	< 0,001	< 0,001	< 0,001	< 0,001	< 0,001	< 0,001
	Reteste	Corr (r)	−0,816	−0,835	−0,580	−0,801	−0,806	−0,720	−0,771
		Valor de *p*	< 0,001	< 0,001	< 0,001	< 0,001	< 0,001	< 0,001	< 0,001
Dia a Dia	Teste	Corr (r)	−0,782	−0,781	−0,566	−0,717	−0,781	−0,661	−0,658
		Valor de *p*	< 0,001	< 0,001	< 0,001	< 0,001	< 0,001	< 0,001	< 0,001
	Reteste	Corr (r)	−0,704	−0,724	−0,466	−0,636	−0,718	−0,694	−0,671
		Valor de *p*	< 0,001	< 0,001	< 0,001	< 0,001	< 0,001	< 0,001	< 0,001
Função	Teste	Corr (r)	−0,855	−0,859	−0,623	−0,814	−0,844	−0,721	−0,764
		Valor de *p*	< 0,001	< 0,001	< 0,001	< 0,001	< 0,001	< 0,001	< 0,001
	Reteste	Corr (r)	−0,821	−0,826	−0,576	−0,765	−0,813	−0,721	−0,748
		Valor de *p*	< 0,001	< 0,001	< 0,001	< 0,001	< 0,001	< 0,001	< 0,001
HHSmBr	Teste	Corr (r)	−0,891	−0,906	−0,617	−0,878	−0,878	−0,801	−0,828
		Valor de *p*	< 0,001	< 0,001	< 0,001	< 0,001	< 0,001	< 0,001	< 0,001
	Reteste	Corr (r)	−0,884	−0,896	−0,602	−0,861	−0,872	−0,796	−0,821
		Valor de *p*	< 0,001	< 0,001	< 0,001	< 0,001	< 0,001	< 0,001	< 0,001

Abreviações: WOMAC, Western Ontario and McMaster Universities; HOOS, Hip Disability and Osteoarthritis Outcome Score; HHSmBr, versão em português do Brasil do Harris Hip Score modificado; Corr(r), correlação.

## Distribuição da Validade de Conteúdo (Piso/Teto)

Nenhum paciente obteve pontuação máxima e/ou mínima durante o teste e reteste do HHSm, somente no domínio DOR tivemos índice próximo dos 30%, com escores de 23,2% e 12,1% no teste e reteste, respectivamente.

## Discussão

O objetivo do presente estudo foi validar o questionário HHS modificado previamente traduzido e adaptado culturalmente para a língua portuguesa. 7 O HHSmBr demonstrou níveis aceitáveis de consistência interna na avaliação de pacientes com diferentes doenças na região do quadril, levando em consideração o alfa de Cronbach que foi de 0,72. Esse resultado corrobora com o estudo de validação na versão árabe do HHSm que apresentou o índice de alfa de Cronbach de 0,72 20 e com os estudos de validação das versões originais do HHS, que apresentaram consistência interna de 0,7, 0,81, 0,94, para as versões turca, italiana e eslovena, respectivamente. 12 16 17

Os resultados mostraram que a versão brasileira do questionário tem propriedades de medidas adequadas, sendo que a confiabilidade teste-reteste foi excelente, com um CCI de 0,80, variando de 0,84 a 0,96. Para a versão original do HHS, os valores de DMCI variam de 15,9 a 18 pontos. 21 No presente trabalho, verificou-se uma DMCI do HHSmBr que varia de 6,60 a 7,37 pontos, o que vai ser útil na aplicação deste questionário em ensaios clínicos, estudos que avaliem efeito de intervenção, já que o DMCI expressa a percepção clínica de melhora do próprio paciente.

Na literatura recente, a validade do HHS tem sido investigada, determinando sua relação com os resultados relatados pelo paciente em outros questionários, tais como o *Short Form-36 Health Survey* (SF-36), o *Total Functional Score* , o *Nonarthritic Hip Score* e o WOMAC. 6 7 12 Em nosso estudo, comparamos os resultados do HHSmBr com os fr outros questionários já validados na língua portuguesa, o WOMAC e HOOS. Ao correlacionar os escores do HHSmBr com os demais escores dos questionários citados verificou-se que o HHSmBr é altamente correlacionado com o WOMAC (r = −0,891) e com o HOOS (r = −0,906). O mesmo aconteceu com os domínios do HHSmBr *,* sendo que o domínio com menor correlação foi o “dia a dia” tanto com o WOMAC como com o HOOS (r = −0,782 e r = −0,781, respectivamente). 22

Os estudos de validação da versão árabe do HHSm incluíram amostras com 80, 16 103, 17 42 12 e 183 pacientes. 20 Para determinar nossa amostra, tomamos como base a maioria dos estudos e a lista de verificação do COSMIN, que considera como excelente o número adotado nesse estudo de 100 pacientes. 10 18

Este estudo tem algumas limitações, como o fato de não ter sido realizado um controle específico da variável cognitiva na inclusão dos pacientes. Levamos em consideração apenas o nível de escolaridade dos pacientes; entretanto, nenhuma dificuldade de compreensão em responder as questões foi percebida e/ou relatada pelo paciente, o que indica que essa variável pode não ter interferido consideravelmente nos resultados.

O HHSm é considerado um questionário de auto-relato; porém, pela logística do serviço, tornou-se inviável esse modelo de aplicação. Dessa forma, optou-se por realizar a aplicação do questionário por via telefônica, assim como em outros trabalhos já realizados. 23 24 25 Além disso, foi garantido com que a aplicação no teste e no reteste fosse feita pelo mesmo examinador, seguindo o rigor de aplicação por telefone. Os mesmos métodos foram usados em ambos os momentos e isso pode ser constatado nos resultados positivos obtidos neste estudo, o que demonstra esse ser um método efetivo e viável de aplicação de questionários.

## Conclusão

O presente estudo mostrou que o HHSmBr é válido e confiável para ser usado na língua portuguesa.
